# The European Food Regulatory Environment Index: a tool to monitor progress in implementing food environment policies

**DOI:** 10.1093/eurpub/ckab106

**Published:** 2021-12-21

**Authors:** Joana Madureira Lima, Mike Rayner, João Breda, Jo Jewell

**Affiliations:** 1 Department of Sociology, University of Oxford, Oxford OX1 1JD, UK; 2 Centre on Population Approaches for Non-Communicable Disease Prevention, Nuffield Department of Population Health, University of Oxford, Oxford OX3 7LF, UK; 3 WHO European Office for the Prevention and Control of Noncommunicable Diseases (NCD Office), Moscow 125009, Russian Federation; 4 WHO Regional Office for Europe, Division of Non Communicable Diseases and Promoting Health through the Lifecourse, Copenhagen DK-2100, Denmark

## Abstract

**Background:**

Evidence based health policy, such as that put forward in the European Food and Nutrition Action Plan 2015–2020 and the WHO Global Action Plan on the Prevention and Control of Noncommunicable Diseases, has a role in curbing the consumption of unhealthful foods and drink. We ask how countries are performing in the adoption of these policies and how the comprehensiveness of their food environment policies explains variations in consumption of unhealthful products across Europe.

**Methods:**

In order to assess the state of policy adoption, we developed a composite indicator—the Food Regulatory Environment Index (FREI) for which we calculated unweighted and weighted formulations according to the strength of the evidence base. We used linear regression models to explain variations in the consumption of unhealthful products as well as variations in a composite indicator of obesogenic diets.

**Results:**

Overall, wealthier countries in the Region perform better. The weighting of the constituent policies does not affect the rankings. We find negative associations between unweighted and weighted formulations of the Index and household consumption of sugary and carbonate drinks as well as with the composite indicator for obesogenic diets.

**Conclusions:**

The main strength of this study is the comprehensiveness and comparability of the policy data across the relatively large number of countries covered. There is a negative association that is statistically significant, between all formulations of the FREI and the household consumption of sugary and carbonated drinks. There is also a negative association between some FREI formulations and obesogenic diets.

## Introduction

Patterns of food consumption and the composition of diets in the World Health Organization (WHO) European Region have changed rapidly in recent decades.^1^^,^^2^ Consumption of processed foods high in saturated fat, free sugars and salt (HFSS foods) is now high across the Region. The shift in fat consumption away from saturated fats and trans-fats to unsaturated fats, and towards the elimination of industrial trans-fats is an important step towards a healthy diet. In a healthy diet, intake of saturated fats should be less than 10% of total energy intake and trans-fats to less than 1% of total energy intake.^3–7^ Limiting intake of free sugars to less than 10% of total energy intake is also part of a healthy diet as is keeping salt intake to less than 5 g per day.^8^ In spite these recommendations, a study of 19 countries in the Region showed that the median average household availability amounted to 33.9% of total purchased dietary energy for unprocessed or minimally processed foods, 19.6% for processed foods and 26.4% for ultra-processed foods. The average household availability of purchased dietary energy from ultra-processed foods ranged from 10.2% in Portugal and 13.4% in Italy to 46.2% in Germany and 50.4% in the UK.^1^ Dietary risk factors characterized by excess caloric intake, saturated fat, free sugar and salt, and low intakes of fruit, vegetables and wholegrain, contribute to hypertension and cardiovascular diseases, Type 2 Diabetes and some types of cancer, leading causes of mortality and morbidity in the WHO European region.^9^^,^^10^ What is more, the prevalence of overweight and obesity in the Region has been increasing steadily to alarming levels: in 2016, 59% of the adult population of Europe was overweight.^11^ The picture is as concerning for children.^12^

The causes of the obesity epidemic are multifactorial in nature and so should be the response to it.^13^^,^^14^ Accordingly, the concept of ‘food environments’ as a range of factors at different levels that shape diets has gained traction among researchers and policy-makers. A food environment approach underlines the important role of governments in developing and implementing policies to ensure that the environments in which we live, work and play are supportive of healthy choices and that manufacturers, retailers and advertisers produce, sell and promote food in a responsible way.

The former includes a set of priority policies based on state-of-the-art knowledge on the factors that influence dietary behaviour and the evidence accumulated on effective approaches (see Supplementary table 1A). The latter outlines policy ‘best buys’ and recommended interventions, including marketing restrictions, front of pack labelling, salt reduction and taxes on sugar-sweetened beverages. Countries in the Region have made use of these frameworks to develop and implement a range of mandatory and voluntary policies, including a growing number of interpretative nutrition labelling schemes, targeted food and beverage taxes, comprehensive reformulation strategies, and restrictions on the marketing of unhealthy foods. Nevertheless, the quality of policy formulation and implementation varies widely across the Region and the status quo in many countries is unlikely to be sufficient to achieve change at scale.^15^^,^^16^

In 2016–2017, WHO led a nutrition policy review process to document the presence of key policies in countries the Region using on a survey sent to all 53 member states to obtain standardized and validated national policy information. Progress was evident in key areas, such as school food standards and fiscal measures, with more countries adopting policies relative to baseline.^11^ However, there were also a number of major gaps in policy implementation, including interpretative front of pack labelling and comprehensive restrictions on food marketing to children.

Composite indicators have been used to monitor policy progress and benchmark suites of policies in the areas of alcohol^17^^,^^18^ and tobacco control policy^19^^,^^20^ allowing countries to measure their relative progress in formulating and adopting policies across the recommended areas of control of these substances. Furthermore, they have been used to explain variations in the consumption of alcohol and tobacco across countries.^21^ We put forward a new composite indicator—the Food Regulatory Environment Index (FREI)—that brings together policies from the different recommended areas to characterize the quality of the regulatory environment in a given country. The FREI aims at helping researchers and policy-makers answer the following questions:

- Which countries in have the most comprehensive set of policies to create healthy food environments?
- How much progress have countries made towards improving food environments against recommended policies?
- Do countries with more comprehensive implementation of healthy food environment policies show lower levels of consumption of different groups of obesogenic foods and drinks at the household level? Do they show lower levels of obesogenic diets in the population?

In this paper, we describe the development of the FREI, and its value in explaining differences in the consumption of obesogenic foods and drinks across the Region.

## Methods

### Data sources

The national level indicators on the state of policies to improve food environments were collected by the WHO Regional Office for Europe in the course of the 2nd Global Nutrition Policy Review (GNPR2), conducted during 2016–2017.^22^ The GNPR2 questionnaire is a comprehensive survey designed by WHO to gauge whether countries have nutrition policies and programmes, how they are being implemented, what the implementation coverage is, who the stakeholders are and how they are coordinated, monitored and evaluated; the questionnaire was also designed to assist WHO report on implementation of key policy frameworks such as the WHO European Food and Nutrition Action Plan. The questionnaire is global in scope, and was circulated to all Member States of the WHO via its regional offices. A list of the questions extracted from the survey and further methodological details on the GNPR2 can be found in the Supplementary table 1A.

Market data for household consumption (Kgs/Household/Year) based on sales data of products such as sugar and chocolate confectionery, biscuits, savoury snacks, soft and carbonated drinks comes from Euromonitor International. Euromonitor collects sales data on these products for 38 countries of the 50 countries in our sample, leaving us with a final sample of 37 countries for which we have both food policy and household consumption data. The distribution of consumption of these products in the population is a truncated, skewed variable; therefore, we performed a logarithmic on transformation the consumption data for each of the items.

We created a measure of Obesogenic Household Diet by normalizing the values for consumption of each of the food and drink products—sugar and chocolate confectionery, biscuits, savoury snacks, soft and carbonated drinks—using z-scores and then applying factor analysis to create a composite indicator.

Our models adjust for differences in percentage of GDP annual growth, urban population as a percentage of the total population, and female labour force participation as a percentage of the workforce. Data for 2016 were sourced from the World Bank Development Indicators Database. GDP annual growth was included because country income drives the nutrition transition.^23^ Similarly, urbanization is associated with a number of risk factors for non-communicable diseases (NCDs).^24^ Female labour force participation was included because of women’s traditional roles cooking in families. There is international evidence, including from some countries in the Region, of an association between hours spent at work by mothers and higher consumption of processed foods.^25–27^

### Building the FREI

We calculated three formulations of the FREI. In the first one, all policies were weighed equally (FREI_ Food Policy). In the second and third formulations, policies were weighted according to the evidence base supporting them as appraised by two different sources (FREI_Food Policy_Informas and FREI_Food Policy_Mozzaffarian). Policies with a stronger evidence base were given more prominence than policies with no or little evidence base.^28^

Policy responses were binary—policy is either present or absent and coded one and zero, respectively. For the non-weighted formulation (FREI_Food Policy), policies were aggregated around sub-indicators that corresponded to recommended policy actions contained in the WHO European Food and Nutrition Action Plan and subsequently in the questionnaire—policies, strategies and plans relevant to nutrition; coordination mechanisms; school health and nutrition programmes; dietary guidelines; nutrition labelling; nutrient declarations; front of pack labelling; menu labelling; nutrition and health claims regulations; reformulation strategies; elimination of trans-fatty acids; control of portion sizes; fiscal policies and taxation; restrictions on marketing of food and non-alcoholic beverages to children; and media campaigns. We calculated the average score for the policy in each sub-indicator (Supplementary table 1A). The sub-indicators were aggregated using a linear aggregation method. In other words, each sub-indicator was given the same weight.

We created a second and a third formulation of the FREI this time attributing weights to each policy according to the level of scientific evidence underpinning it. For the second formulation, the basis of the weighing system was the American Heart Association (AHA) Scientific Statement Population Approaches to Improve Diet, Physical Activity and Smoking Habits^29^ (FREI_ Food Policy_Mozzaffarian). We attributed the authors’ weight to each one of the policies collected by the survey. When the authors had not considered one of our chosen polices, e.g. those pertaining to the promotion and protection breastfeeding, we used alternative sources of evidence and classified according to the AHA Classification of policies based on the Recommendations and Level of Evidence for Population Level model.^29^ We attributed weights on a 0–9 scale. 0 corresponds to the lowest level of evidence—Class III and level of evidence C (‘There is evidence and/or general consensus that the intervention is not useful/effective and in some cases may be harmful and the weight of evidence is supported by only consensus of opinion of experts, case studies, standard of care, etc.’) and 9 corresponds to Class I Level of Evidence A (‘There is evidence for and/or general agreement that the intervention is beneficial, useful and effective. The intervention should be performed/data derived from multiple RTCs.’) Further information in the Supplementary File 2. Where we knew new evidence had surfaced since 2012, the year the scientific statement came out, we revisited the classification of the policy in question.

For the third formulation of the FREI (FREI_Food Policy_Informas), we used an alternative set of weights proposed by Mahesh and colleagues. Weightings in this formulation resulted from a two-round Delphi study to determine the relative contributions of 19 widely recommended good practice food environment policies to improve population nutrition, based on evidence of effectiveness and expert ratings. The purpose of determining the weighting was to facilitate benchmarking of the implementation of food environment policies globally.^30^ For example, a ‘High tax on unhealthy foods’ receives twice the weight of ‘Private workplace food policies’.

The resulting scores for the Index were rescaled to a 1–100 scale, with higher scores indicating a more comprehensive combination of policies in place. The benchmark country is 100. Under the weighted scenarios, the country with the most comprehensive combination of ‘highly-weighted’ policies is the benchmark country.

### Analysis

In order to quantify the association between policy comprehensiveness and consumption of obesogenic foods and drinks, we used a linear regression model. Firstly, the FREI scores were used to test the hypothesis that more comprehensive regulatory environments are associated with lower consumption of obesogenic foods and drinks at the household level—sugar and chocolate confectionery, biscuits, savoury snacks, soft and carbonated drinks. Secondly, we tested the hypothesis that more comprehensive regulatory environments are associated with a lower prevalence of an obesogenic diet as proxied by a composite measure. We adjusted both models for GDP Annual Growth of the Population living in Urban Areas and for Geographic Sub region (e.g. Nordic countries, Southern Countries, Western Countries).

## Results

**Figure 1:**
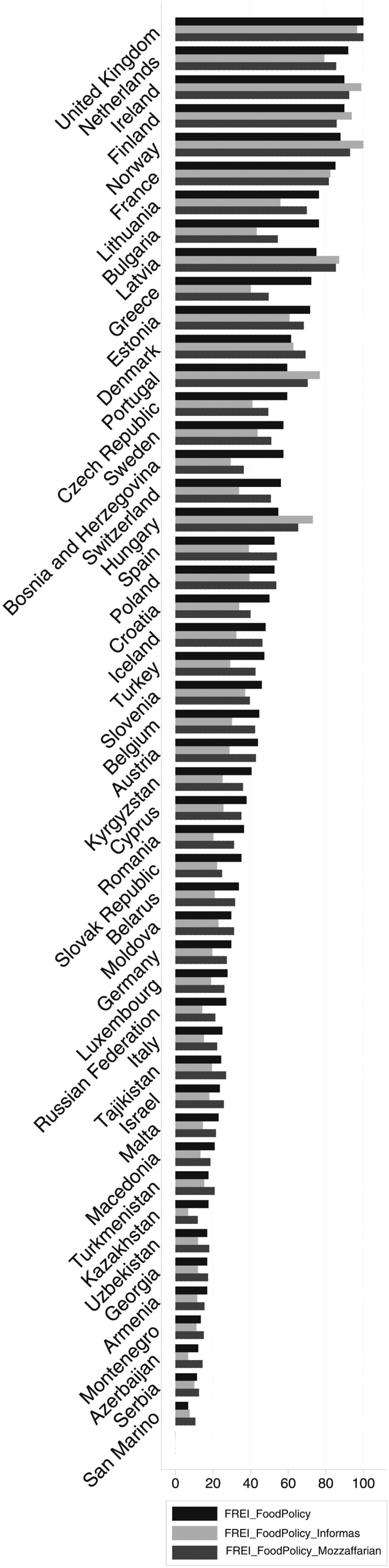
FREI scores according to different formulations

The weighted and non-weighted formulations are highly correlated as are the rankings of countries according to each formulation (figure 1). There isn’t a clear-cut geographic clustering of the ranks but, overall, wealthier countries in the Region, such as the United Kingdom, Ireland and Norway, tend to perform better (Germany and Luxembourg are notable exceptions scoring low across weighted and non-weighted formulations) and poorer countries in the South and East of the region tend to score less well. Applying weighting to the policies contained in the index does not result in major changes to the overall ranking of countries. Ireland, Norway, Latvia and Hungary perform marginally better under the weighting proposed by Mahesh and colleagues, likely due to the weight given to fiscal measures.

When we use the FREI to explain variations in the consumption of each of the products, the unadjusted models do not show any obvious associations (table 1). However, once we adjust for the geographic region of the country, GDP Annual Growth, urbanization and female labour force participation, different patterns emerge. There is a negative association, that is statistically significant, between all three formulations of the FREI and the household consumption of sugary drinks and carbonated drinks. For each unit increase in the non-weighted FREI (FREI_Food Policy), there is a reduction of 2.15 l of sugary drinks per year per household 95% CI [−4.10; −0.19] and a reduction of 1.05 l of carbonate drinks per year per household 95% CI [−1.90; −0.21] (table 1). The associations between the Index and sports drinks are also negative but not statistically significant in any of the formulations (table 1 and Supplementary tables 3SF and 5SF).

**Table 1:** Linear regression models of household sugary drink consumption on FREI scores

	(1)	(2)	(3)	(4)	(5)	(6)
Variables	Soft drinks—Litres per household	Soft drinks—Litres per household (Logged)	Sports energy drinks—Litres per household	Sports energy drinks—Litres per household (Logged)	Carbonates—Litres per household	Carbonates—Litres per household (Logged)
Food Regulatory Environment Index	−2.148^**^	−0.00579^**^	−0.0325	−0.00585	−1.046^**^	−0.00840^**^
	(0.952)	(0.00256)	(0.0500)	(0.00789)	(0.408)	(0.00315)
Sub Regions of Europe						
Central Eastern (Reference)						
Central Western	−11.80	0.0683	3.506	−0.353	−16.05	−0.0950
	(88.71)	(0.239)	(4.655)	(0.735)	(38.00)	(0.294)
Eastern	−151.3^**^	−0.515^***^	−4.138	−1.477^**^	−8.933	−0.164
	(66.33)	(0.178)	(3.481)	(0.549)	(28.42)	(0.220)
Nordic	−146.9^*^	−0.325	0.139	−0.187	4.545	0.0417
	(77.76)	(0.209)	(4.081)	(0.644)	(33.31)	(0.257)
South Eastern	60.25	0.184	−1.249	−0.350	23.09	0.158
	(59.23)	(0.159)	(3.108)	(0.491)	(25.37)	(0.196)
Southern	73.93	0.189	−0.275	−0.495	−14.30	−0.164
	(71.74)	(0.193)	(3.765)	(0.594)	(30.73)	(0.237)
Western	50.52	0.143	7.199^*^	0.369	12.20	0.0702
	(72.76)	(0.196)	(3.818)	(0.603)	(31.17)	(0.241)
GDP Annual Growth (%)	3.841	0.00704	0.665^**^	0.0764^*^	5.927^**^	0.0370^**^
	(5.142)	(0.0138)	(0.270)	(0.0426)	(2.203)	(0.0170)
Urban population % of total population	5.539^**^	0.0120^*^	0.128	0.0279	2.723^***^	0.0205^***^
	(2.172)	(0.00584)	(0.114)	(0.0180)	(0.931)	(0.00719)
Female labour force participation	−8.131	−0.00717	0.525	0.0691	2.328	0.0166
	(6.376)	(0.0171)	(0.335)	(0.0528)	(2.731)	(0.0211)
Constant	536.1^*^	5.819^***^	−23.63	−2.742	−107.9	3.123^***^
	(292.0)	(0.785)	(15.32)	(2.419)	(125.1)	(0.966)

Observations	38	38	38	38	38	38
R-squared	0.657	0.629	0.551	0.528	0.445	0.430

Note: Standard errors in parentheses.

*** *P* < 0.01,

** *P* < 0.05,

* *P* < 0.1.

**Table 2:** Linear regression models of sugary confectionery and savoury snacks consumption on FREI scores

Variables	(1)	(2)	(3)	(4)	(5)	(6)	(7)	(8)
	Sugar confectionery—Kgs per household	Sugar confectionery—Kgs per household (Logged)	Choco confectionery—Kgs per household	Choco confectionery—Kgs per Household (Logged)	Biscuits—Kgs per household	Biscuits—Kgs per household (Logged)	Savoury snacks—Kgs per household	Savoury snacks—Kgs per household (Logged)
Food Regulatory Environment Index	−0.0382^*^	−0.00771^*^	−0.0491^*^	−0.00783^**^	−0.0354	−0.0354	−0.0476	−0.00657^*^
	(0.0191)	(0.00429)	(0.0274)	(0.00319)	(0.0371)	(0.0371)	(0.0383)	(0.00358)
Sub Regions of Europe								
Central Eastern (Reference)								
Central Western	2.005	0.405	3.212	0.187	7.292^**^	7.292^**^	7.773^**^	0.688^**^
	(1.781)	(0.399)	(2.552)	(0.297)	(3.459)	(3.459)	(3.568)	(0.333)
Eastern	3.511^**^	0.515^*^	−2.730	−0.453^*^	1.689	1.689	−0.770	−0.318
	(1.332)	(0.299)	(1.909)	(0.222)	(2.587)	(2.587)	(2.668)	(0.249)
Nordic	5.957^***^	0.910^**^	0.930	−0.0485	−1.051	−1.051	8.395^**^	0.681^**^
	(1.561)	(0.350)	(2.238)	(0.261)	(3.032)	(3.032)	(3.128)	(0.292)
South Eastern	−1.142	−0.496^*^	−2.537	−0.211	1.745	1.745	1.677	0.169
	(1.189)	(0.267)	(1.704)	(0.199)	(2.310)	(2.310)	(2.382)	(0.223)
Southern	−0.911	−0.467	−5.239^**^	−0.794^***^	3.291	3.291	3.768	0.232
	(1.441)	(0.323)	(2.064)	(0.240)	(2.797)	(2.797)	(2.885)	(0.270)
Western	3.371^**^	0.551	3.434	0.0890	0.791	0.791	1.307	0.128
	(1.461)	(0.327)	(2.094)	(0.244)	(2.837)	(2.837)	(2.926)	(0.274)
GDP Annual Growth (%)	0.175	0.0226	0.235	0.0142	0.00704	0.00704	0.598^***^	0.0267
	(0.103)	(0.0231)	(0.148)	(0.0172)	(0.200)	(0.200)	(0.207)	(0.0193)
Urban population % of total population	0.0544	0.0132	0.0426	0.0134^*^	0.211^**^	0.211^**^	0.184^**^	0.0171^**^
	(0.0436)	(0.00978)	(0.0625)	(0.00728)	(0.0847)	(0.0847)	(0.0874)	(0.00817)
Female labour force participation	0.147	0.0161	0.284	0.0352	−0.797^***^	−0.797^***^	0.134	0.00777
	(0.128)	(0.0287)	(0.183)	(0.0214)	(0.249)	(0.249)	(0.256)	(0.0240)
Constant	−4.744	0.0972	−2.315	0.353	33.39^***^	33.39^***^	−8.874	0.888
	(5.863)	(1.314)	(8.402)	(0.979)	(11.39)	(11.39)	(11.74)	(1.098)

Observations	38	38	38	38	38	38	38	38
R-squared	0.706	0.661	0.622	0.587	0.609	0.609	0.671	0.610

Note: Standard errors in parentheses.

*** *P* < 0.01,

** *P* < 0.05,

* *P* < 0.1.

As for the consumption of confectionary and savoury snacks, we observe a different picture. We find negative associations between the non-weighted FREI (FREI_Food Policy) scores and the consumption of sugar and chocolate confectionery, but they are not significant at the 95% level (table 2). We do not find statistically significant associations for household consumption of biscuits nor savoury snacks. Similarly, for the weighted indices (FREI_Food Policy_Informas and FREI_Food Policy_Mozzaffarian), we do not find any statistically significant coefficients for the associations between the different formulations of the FREI and the household consumption of sugar or chocolate confectionery, biscuits nor savoury snacks (Supplementary tables 2SF and 3SF).

Finally, when we calculate the associations between the non-weighted and weighted formulations of the FREI and the composite indicator for obesogenic diet, we observe different associations. Using the non-weighted formulation (FREI_Food Policy), we find that there is a very mild association between countries’ score on the Index and the obesogenic composition of the diet. In other words, for each unit increase in the FREI scores we see 0.01 unit increase (95%CI [−0.01; −0.00]) in the obesogenic diet composite indicator. As for the remaining formulations, we do not find statistically significant associations. (Supplementary tables 2SF and 3SF).

## Discussion

Our study introduces a novel tool to help countries assess their relative position in terms of the comprehensiveness of their food environment policies. We find that there is not a clear-cut geographic clustering to the ranking of countries but, overall, wealthier countries in the Region tend to perform better than poorer ones. The weighting of the constituent policies according to the strength of the evidence base underpinning them does not affect the rankings in a substantive way. We find negative associations between policy comprehensiveness as measured by the Index and the household consumption of sugary and carbonate drinks.

Other composite measures to monitor or assess the degree of implementation of widely recommended policies and actions have been used in country comparisons. Notably, INFORMAS (The International Network for Food and Obesity/NCDs Research, Monitoring and Action Support) has developed the ‘Healthy Food Environment Policy Index’ (Food-EPI) to assess and benchmark the extent of implementation of policies for creating healthy food environments by national governments.^31^^,^^32^ More recently, as described in Mahesh et al, INFORMAS used the Delphi methodology and Analytic Hierarchy Process to derive weightings for each of the policy components of the index based on their relative contribution. A recent publication summarized the results of the Food-EPI when applied in 11 countries across 6 regions.^31^ Other work, such as that by Mozzafarian et al., has attempted to grade policies based on their benefit, usefulness and effectiveness as well as the quality of the underpinning evidence. The main comparative advantage and added value of our study is the comprehensiveness and comparability of the GNPR2 data across a large number of countries and broad range of policies that influence dietary behaviours. Compared with previous work examining policy implementation in countries, we have large geographical reach, greater coverage of non-English speaking countries and data that has been validated by country experts and WHO staff.

Our study has a number of limitations, not least the level of accuracy in country reporting. Efforts were made to eliminate discrepancies between what was reported by countries and the information available from other published resources. When discrepancies arouse, we contacted the national focal point for the survey for clarification. Further, important details and nuances in policies can unavoidably be lost when comparing against a common policy typology and across many different policy areas. For example, the details of criteria adopted—such as in labelling, school food or marketing policies—may have a large impact on the policy implementation and potential for public health benefit, our Index may not be able to fully account for those nuances as it relies on a binary (Y/N) approach. Lastly, sales data was only available for 38 out of the 50 countries for which we had policy data. The inclusion of the remaining countries in future analyses could shed more light on these associations.

Despite the limitations, this Index can make a substantial contribution to the literature on policy implementation. Overall, our results show that more comprehensive combinations of policies are associated with reductions in the household consumption of obesogenic foods and drinks. However, the negative association does not emerge consistently across all products. One plausible explanation is that sugary drinks and carbonates, where associations are negative, are more frequently targeted by or covered by existing policies in countries, and/or the policies that apply to them more often explicitly aim to reduce purchase and consumption. For example, in all instances where a country reports health-related fiscal measures such as a tax, sugary drinks and carbonates are subject to that policy.^33^

Food reformulation and front of pack labelling are examples of policies that might apply to the food categories under study. Here, the objective of the policy is not always to reduce purchase and consumption, rather to improve the nutritional composition and shift purchases towards healthier options (typically within the same category), as such the impact on sales or household consumption might not be so great but an important impact on nutrient intake may be observed.^34^ As such, changes to nutritional composition/portion sizes of food may help capture the impact more fully; increasingly commercial food purchase data is available for such purposes but was beyond the scope of this paper.^35^ In addition, it must be said that not all countries have rigorously implemented these policies—with patchy reformulation and low uptake of labelling schemes, generally under voluntary conditions.^36^ Such factors may in part explain the different findings between the product categories and point to the need to consider regulatory approaches.

There are also important contextual factors that can, in part, explain why some countries perform less well and appear to have made limited progress. On the one hand, governments operate within a political environment and may feel that their mandate or scope to drive such a suite of policies is limited and the food industry may also vocally oppose some of the proposed policies.^37^ This is not the case universally, and some countries have managed to overcome significant barriers. However, not all governments currently have equal technical, financial and human resource capacity to design and manage policies, nor do they have the same ability to hold industry to account.

Another explanation could lie on the fact that the more developed and ‘mature’ the diet-related NCD and obesity epidemic, the higher the likelihood that stricter policies will have been introduced. For example, the UK has had one of the highest rates of obesity for a long time, and has been through several rounds of obesity and diet policy development processes to arrive at the current basket of policies that contributed to its high score.^38^ Countries that are earlier in their overweight and obesity epidemic may have less of a sense of urgency than countries with much more established epidemics, and may have been focusing their efforts on other dimensions of malnutrition (e.g. Central Asian and Caucasus regions). However, these countries also report some of the highest sodium intakes in the world and extreme levels of salt and industrial trans-fatty acids in food, meaning that the need for policy action to promote healthy diets is no less pressing.^16^ In addition, in the WHO European region, the highest rates of childhood obesity are observed in southern European countries and they do not currently perform as well as northern European countries. Mediterranean countries perform poorly in this index. This is despite indications that that traditional Mediterranean diet is under serious pressure in these countries, and risks being undermined by diets high in HFSS foods.^39^ In this way, the FREI can serve to highlight where additional action in countries might be needed.

## Supplementary data

Supplementary data are available at *EURPUB* online.

## Funding

Joana Madureira Lima was the recipient of a doctoral scholarship form the Portuguese Foundation for Science and Technology.

*Conflicts of* *interest:* None declared.

**Key points**

- The Food Regulatory Environment Index (FREI) is a novel tool to study the quality of food environments across Europe.
- Wealthier countries in Europe obtain higher ranks in the FREI.
- Household consumption of sugary and carbonate drinks is negatively associated with the FREI scores.
- There is also a negative association between some FREI formulations and obesogenic diets.

## Supplementary Material

ckab106_Supplementary_Data
