# Supplementary material for: The European Food Regulatory Environment Index: a tool to monitor progress in implementing food environment policies
Source: Eur J Public Health. 2021 Dec 21;32(2):261–6. doi: 10.1093/eurpub/ckab106 (PMC8975533; doi:10.1093/eurpub/ckab106)
Supplement: ckab106_Supplementary_Data [file ckab106_supplementary_data.zip › ckab106-suppl_data/ejph-2020-10-om-1243-File003.docx]

Supplementary File

Table 1SF Subcomponents and variables in the GNPR2 survey

| **Subcomponent** | **Variable** |  |  |  |  |  |  |  |  |  |  |  |  |  |  |  |  |  |  |  |  |
| --- | --- | --- | --- | --- | --- | --- | --- | --- | --- | --- | --- | --- | --- | --- | --- | --- | --- | --- | --- | --- | --- |
| Policies, Strategies and Plans Relevant to Nutrition | *Are there national nutrition policies, strategies or action plans ot other policies and plans (e.g. health sector plans, social protection plan,*  *food security strategies) which focus on healthy diets?* | | | | | | | | | | | | | | | | | | |  |  |
|  |  |  |  |  |  |  |  |  |  |  |  |  |  |  |  |  |  |  |  |  |  |
| Coordination Mechanisms for Nutrition | *Are there established coordination mechanisms for nutrition?* | | | | |  |  |  |  |  |  |  |  |  |  |  |  |  |  |  |  |
|  |  |  |  |  |  |  |  |  |  |  |  |  |  |  |  |  |  |  |  |  |  |
| Actions related to Maternal Infant and Young Child Nutrition | *Is growth monitoring and promotion for children less than 5 years of age being implemented?* | | | | | | | |  |  |  |  |  |  |  |  |  |  |  |  |  |
|  | *Is breastfeeding promotion and counselling being implemented?* | | | | |  |  |  |  |  |  |  |  |  |  |  |  |  |  |  |  |
|  | *Is the Baby Friendly Initiative being Implemented?* | | | |  |  |  |  |  |  |  |  |  |  |  |  |  |  |  |  |  |
|  | *Is complementary feeding counselling or education being implemented?* | | | | | |  |  |  |  |  |  |  |  |  |  |  |  |  |  |  |
| School Health and Nutrition | *Are school health and nutrition policies, programmes or related standards being implemented?* | | | | | | | |  |  |  |  |  |  |  |  |  |  |  |  |  |
|  | *Are all children in the target schools eligible to participate in the school fruit and vegetable scheme?* | | | | | | | |  |  |  |  |  |  |  |  |  |  |  |  |  |
|  | *Are all children in the targeted schools eligible to participate in the school milk scheme?* | | | | | | |  |  |  |  |  |  |  |  |  |  |  |  |  |  |
|  |  |  |  |  |  |  |  |  |  |  |  |  |  |  |  |  |  |  |  |  |  |
| Dietary Guidelines | *Are there national dietary guidelines?* | | |  |  |  |  |  |  |  |  |  |  |  |  |  |  |  |  |  |  |
|  | *What kinds of dietary guidelines exist in your country?* | | | | |  |  |  |  |  |  |  |  |  |  |  |  |  |  |  |  |
|  | *Do the nutrient based dietary guidelines exist for different target groups?* | | | | | |  |  |  |  |  |  |  |  |  |  |  |  |  |  |  |
|  | *Do the food based dietary guidelines exist for different target groups?* | | | | | |  |  |  |  |  |  |  |  |  |  |  |  |  |  |  |
|  |  |  |  |  |  |  |  |  |  |  |  |  |  |  |  |  |  |  |  |  |  |
| Nutrition Labelling | *Are there any actions being taken to regulate to guide the implementation of nutrition labelling on pre-packaged foods?* | | | | | | | | | |  |  |  |  |  |  |  |  |  |  |  |
|  |  |  |  |  |  |  |  |  |  |  |  |  |  |  |  |  |  |  |  |  |  |
| Nutrient Declaration | *Is nutrient declaration mandatory?* | | |  |  |  |  |  |  |  |  |  |  |  |  |  |  |  |  |  |  |
|  |  |  |  |  |  |  |  |  |  |  |  |  |  |  |  |  |  |  |  |  |  |
| Front of Pack Labelling | *Is front of pack labelling mandatory or voluntary on pre-packaged foods and beverages?* | | | | | | |  |  |  |  |  |  |  |  |  |  |  |  |  |  |
|  | *Is front of pack labelling implemented through legislation, regulation or guidelines?* | | | | | | |  |  |  |  |  |  |  |  |  |  |  |  |  |  |
|  | *Is a nutrient profile model used to implement the front of pack labelling systems?* | | | | | | |  |  |  |  |  |  |  |  |  |  |  |  |  |  |
|  |  |  |  |  |  |  |  |  |  |  |  |  |  |  |  |  |  |  |  |  |  |
| Menu Labelling | *Is menu labelling mandatory or voluntary?* | | | |  |  |  |  |  |  |  |  |  |  |  |  |  |  |  |  |  |
|  | *Is menu labelling implemented through legislation, regulations or guidelines?* | | | | | | |  |  |  |  |  |  |  |  |  |  |  |  |  |  |
|  |  |  |  |  |  |  |  |  |  |  |  |  |  |  |  |  |  |  |  |  |  |
| Nutrition and Health Claims | *Are measures to regulate or guide nutrition or health being implemented?* | | | | | |  |  |  |  |  |  |  |  |  |  |  |  |  |  |  |
|  | *Do the nutrition health claims follow the upper level conditions specified in the Codex guidelines for making claims that the product is "low" in or "free"*  *from energy, total fat, saturated fatty acids, cholesterol, sugars and sodium?* | | | | | | | | | | | | | | | | | | | | |
|  | *Do the nutrition health claims follow the upper level conditions specified in the Codex guidelines for making claims that the product is "low" in or "free"*  *in protein, vitamins and minerals and dietary fiber?* | | | | | | | | | | | | | | | | | | |  |  |
|  |  |  |  |  |  |  |  |  |  |  |  |  |  |  |  |  |  |  |  |  |  |
|  |  |  |  |  |  |  |  |  |  |  |  |  |  |  |  |  |  |  |  |  |  |
| Reformulation of foods and beverages | *Are there measures to reformulate foods and beverages which are high in saturated fatty acids, trans fattty acids, sugars or salt?* | | | | | | | | | |  |  |  |  |  |  |  |  |  |  |  |
|  | *Are the reformulation measures to reduce the content of saturated fatty acids mandatory or voluntary?* | | | | | | | |  |  |  |  |  |  |  |  |  |  |  |  |  |
|  | *Have specific reformulation targets been set to reduce the content of saturated fatty acids?* | | | | | | |  |  |  |  |  |  |  |  |  |  |  |  |  |  |
|  | *Is any guidance provided as to what saturated fatty acids are to be replaced with?* | | | | | | |  |  |  |  |  |  |  |  |  |  |  |  |  |  |
|  | *Are the reformulation measures to reduce the content of trans fatty acids mandatory or voluntary?* | | | | | | | |  |  |  |  |  |  |  |  |  |  |  |  |  |
|  | *Are the reformulation measures to reduce the content of sugars mandatory or voluntary?* | | | | | | |  |  |  |  |  |  |  |  |  |  |  |  |  |  |
|  | *Have specific reformulation targets been set to reduce the content of salt/sodium?* | | | | | | |  |  |  |  |  |  |  |  |  |  |  |  |  |  |
|  |  | | | | | | | |  |  |  |  |  |  |  |  |  |  |  |  |  |
|  | *Is a specific measure to ban or virtually eliminate industrial trans fatty acids being implemented?* | | | | | | | |  |  |  |  |  |  |  |  |  |  |  |  |  |
| Elimination of Trans Fatty Acids | *Is there a measure to ban or virtually eliminate industrial trans fatty acids mandatory or voluntary* | | | | | | | |  |  |  |  |  |  |  |  |  |  |  |  |  |
|  | *Is the measure to ban or virtually eliminate industrial fatty acids set forth in any regulation, legislation or guidelines?* | | | | | | | | | |  |  |  |  |  |  |  |  |  |  |  |
|  | *Does the measure to ban or virtually eliminate industrial trans fatty acids apply to all foods or meals?* | | | | | | | |  |  |  |  |  |  |  |  |  |  |  |  |  |
|  |  |  |  |  |  |  |  |  |  |  |  |  |  |  |  |  |  |  |  |  |  |
| Fiscal policies for healthy diets | *Are fiscal policies implemented to reduce the consumption of unhealthy foods and beverages / encourage the consumption of healthier ones?* | | | | | | | | | | |  |  |  |  |  |  |  |  |  |  |
|  |  |  |  |  |  |  |  |  |  |  |  |  |  |  |  |  |  |  |  |  |  |
| Taxation | *Is taxation of different products categories set forth in any legislation, regulation or guidelines?* | | | | | | | |  |  |  |  |  |  |  |  |  |  |  |  |  |
|  |  |  |  |  |  |  |  |  |  |  |  |  |  |  |  |  |  |  |  |  |  |
| Marketing of food and non-alcoholic beverages | *Are measures to regulate or guide marketing of food and non-alcoholic beverages to children being implemented?* | | | | | | | | |  |  |  |  |  |  |  |  |  |  |  |  |
|  | *Are the mandatory or voluntary measures set forth in any legislation, regulation or guidelines?* | | | | | | | |  |  |  |  |  |  |  |  |  |  |  |  |  |
|  | *Do the measures clearly define the age of the children to which the measures apply?* | | | | | | |  |  |  |  |  |  |  |  |  |  |  |  |  |  |
|  | *Do the measures clearly define what is considered "marketing to children"?* | | | | | |  |  |  |  |  |  |  |  |  |  |  |  |  |  |  |
|  | *Do the measures clearly define the marketing techniques covered for each communication channel, setting or context?* | | | | | | | | | |  |  |  |  |  |  |  |  |  |  |  |
|  | *Do the measures clearly define which foods and beverages are covered ?* | | | | | |  |  |  |  |  |  |  |  |  |  |  |  |  |  |  |
|  |  | | | | |  |  |  |  |  |  |  |  |  |  |  |  |  |  |  |  |
| Control of portion sizes | *Are measures to control portion sizes being implemented?* | | | | |  |  |  |  |  |  |  |  |  |  |  |  |  |  |  |  |
|  | *Are the portion control size measures set forth in any legislation, regulations or guidelines?* | | | | | | | |  |  |  |  |  |  |  |  |  |  |  |  |  |
|  |  |  |  |  |  |  |  |  |  |  |  |  |  |  |  |  |  |  |  |  |  |

Table 2SF. Linear regression of Obesogenic Diet Composite Indicator on FREI alternative formulations

|  | (1) | (2) | (3) |
| --- | --- | --- | --- |
| VARIABLES | Obesogenic Diet Composite Indicator | Obesogenic Diet Composite Indicator | Obesogenic Diet Composite Indicator |
| Food Regulatory Environment Index | -0.00743** |  |  |
|  | (0.00285) |  |  |
| FREI (Informas) |  | -0.264* |  |
|  |  | (0.130) |  |
| FREI (Mozzaffarian et al.) |  |  | -0.163* |
|  |  |  | (0.0846) |
| Sub Regions of Europe |  |  |  |
| Central Eastern (Reference) |  |  |  |
| Central Western | 0.432 | 0.690** | 0.653** |
|  | (0.266) | (0.309) | (0.305) |
| Eastern | -0.150 | -0.170 | -0.180 |
|  | (0.199) | (0.229) | (0.234) |
| Nordic | 0.260 | 0.434 | 0.379 |
|  | (0.233) | (0.259) | (0.255) |
| South Eastern | 0.0286 | 0.0232 | 0.00532 |
|  | (0.177) | (0.188) | (0.190) |
| Southern | -0.0155 | 0.0924 | 0.0704 |
|  | (0.215) | (0.223) | (0.225) |
| Western | 0.387* | 0.456* | 0.464* |
|  | (0.218) | (0.229) | (0.230) |
| GDP Annual Growth (Percentage) | 0.0496*** | 0.0521*** | 0.0512*** |
|  | (0.0154) | (0.0165) | (0.0166) |
| Urban Population % of Total Population | 0.0203*** | 0.0149** | 0.0160** |
|  | (0.00651) | (0.00658) | (0.00672) |
| Female Labor Force Participation | 0.0118 | 0.00749 | 0.00510 |
|  | (0.0191) | (0.0200) | (0.0198) |
| Constant | -1.681* | -1.305 | -1.190 |
|  | (0.875) | (0.898) | (0.893) |
| Observations | 38 | 37 | 37 |
| R-squared | 0.690 | 0.663 | 0.659 |

Standard errors in parentheses *** p<0.01, ** p<0.05, * p <0.1

Table 3SF. Linear regression models of household sugary drink consumption on FREI scores (Mozzafarian Scale)

|  | (1) | (2) | (3) | (4) | (5) | (6) |
| --- | --- | --- | --- | --- | --- | --- |
| VARIABLES | Soft Drinks - Litres per Household | Soft Drinks - Litres per Household (Logged) | Sports EnergyDrinks - Litres per Household | Sports EnergyDrinks - Litres per Household (Logged) | Carbonates - Litres per Household | Carbonates - Litres per Household  (Logged) |
|  |  |  |  |  |  |  |
| FREI (Mozzaffarian) | -71.81*** | -0.194*** | -0.651 | -0.196 | -32.71*** | -0.248*** |
|  | (25.66) | (0.0691) | (1.424) | (0.221) | (10.91) | (0.0859) |
| Sub Regions of Europe |  |  |  |  |  |  |
| Central Eastern (Reference) |  |  |  |  |  |  |
| Central Western | 83.11 | 0.325 | 4.364 | -0.0755 | 26.67 | 0.229 |
|  | (92.62) | (0.249) | (5.140) | (0.799) | (39.36) | (0.310) |
| Eastern | -170.6** | -0.572*** | -3.993 | -1.689** | -11.61 | -0.172 |
|  | (71.02) | (0.191) | (3.941) | (0.612) | (30.18) | (0.238) |
| Nordic | -107.5 | -0.216 | 0.532 | -0.00556 | 20.58 | 0.164 |
|  | (77.33) | (0.208) | (4.291) | (0.667) | (32.87) | (0.259) |
| South Eastern | 50.29 | 0.157 | -1.316 | -0.395 | 19.16 | 0.130 |
|  | (57.58) | (0.155) | (3.195) | (0.496) | (24.47) | (0.193) |
| Southern | 85.11 | 0.220 | 0.0590 | -0.417 | -9.375 | -0.119 |
|  | (68.27) | (0.184) | (3.788) | (0.589) | (29.01) | (0.228) |
| Western | 56.29 | 0.161 | 7.475* | 0.448 | 14.20 | 0.0933 |
|  | (69.77) | (0.188) | (3.872) | (0.601) | (29.65) | (0.233) |
| GDP Annual % Growth | 4.506 | 0.00893 | 0.666** | 0.0814* | 6.117*** | 0.0383** |
|  | (5.020) | (0.0135) | (0.279) | (0.0433) | (2.133) | (0.0168) |
| Urban Population % of Total Population | 5.161** | 0.0109* | 0.113 | 0.0234 | 2.586*** | 0.0190*** |
|  | (2.038) | (0.00549) | (0.113) | (0.0176) | (0.866) | (0.00682) |
| Female Labor Force Participation | -8.571 | -0.00831 | 0.491 | 0.0690 | 1.939 | 0.0125 |
|  | (6.013) | (0.0162) | (0.334) | (0.0518) | (2.555) | (0.0201) |
| Constant | 614.4** | 6.034*** | -21.57 | -2.371 | -69.77 | 3.461*** |
|  | (271.0) | (0.730) | (15.04) | (2.336) | (115.2) | (0.906) |
|  |  |  |  |  |  |  |
| Observations | 37 | 37 | 37 | 37 | 37 | 37 |
| R-squared | 0.675 | 0.644 | 0.539 | 0.535 | 0.489 | 0.453 |

Standard errors in parentheses

*** p<0.01, ** p<0.05, * p<0

Table 4SF. Linear regression models of sugary confectionery and savoury snacks consumption on FREI Mozzaffarian et al. formulation

|  | (1) | (2) | (3) | (4) | (5) | (6) | (7) | (8) |
| --- | --- | --- | --- | --- | --- | --- | --- | --- |
| VARIABLES | Sugar Confectionery - Kgs per Household | Sugar Confectionery - Kgs per Household (Logged) | Choco Confectionery- Kgs per Household | Choco Confectionery- Kgs per Household (Logged) | Biscuits- Kgs per Household | Biscuits- Kgs per Household (Logged) | Savoury Snacks - Kgs per Household | Savoury Snacks - Kgs per Household (Logged) |
|  |  |  |  |  |  |  |  |  |
| FREI (Mozzaffarian et al) | -0.0180 | 0.0584 | -0.880 | -0.121 | -0.114 | -0.0590 | -1.176 | -0.160 |
|  | (0.579) | (0.128) | (0.782) | (0.0956) | (1.052) | (0.104) | (1.063) | (0.0977) |
| Sub Regions of Europe |  |  |  |  |  |  |  |  |
| Central Eastern (Reference) |  |  |  |  |  |  |  |  |
| Central Western | 1.987 | 0.322 | 4.478 | 0.357 | 7.531* | 0.898** | 9.473** | 0.919** |
|  | (2.089) | (0.463) | (2.824) | (0.345) | (3.797) | (0.377) | (3.838) | (0.352) |
| Eastern | 4.739*** | 0.802** | -3.355 | -0.479* | 1.600 | 0.0632 | -2.105 | -0.489* |
|  | (1.602) | (0.355) | (2.165) | (0.265) | (2.911) | (0.289) | (2.943) | (0.270) |
| Nordic | 5.928*** | 0.886** | 1.917 | 0.0747 | -0.507 | 0.203 | 9.691*** | 0.854*** |
|  | (1.744) | (0.386) | (2.358) | (0.288) | (3.170) | (0.314) | (3.204) | (0.294) |
| South Eastern | -1.043 | -0.467 | -2.721 | -0.230 | 1.696 | 0.298 | 1.393 | 0.131 |
|  | (1.299) | (0.288) | (1.756) | (0.215) | (2.361) | (0.234) | (2.386) | (0.219) |
| Southern | -0.327 | -0.319 | -4.402** | -0.670** | 4.141 | 0.438 | 4.581 | 0.342 |
|  | (1.540) | (0.341) | (2.081) | (0.254) | (2.799) | (0.278) | (2.829) | (0.260) |
| Western | 3.897** | 0.693* | 4.276* | 0.210 | 1.689 | 0.350 | 2.147 | 0.241 |
|  | (1.574) | (0.349) | (2.127) | (0.260) | (2.860) | (0.284) | (2.891) | (0.266) |
| GDP Annual Growth (5) | 0.156 | 0.0179 | 0.254 | 0.0157 | 0.0136 | -0.00138 | 0.631*** | 0.0309 |
|  | (0.113) | (0.0251) | (0.153) | (0.0187) | (0.206) | (0.0204) | (0.208) | (0.0191) |
| Urban Population % of Total Population | 0.0264 | 0.00564 | -0.00343 | 0.00681 | 0.163* | 0.0112 | 0.138 | 0.0109 |
|  | (0.0460) | (0.0102) | (0.0621) | (0.00759) | (0.0836) | (0.00829) | (0.0845) | (0.00776) |
| Female Labour Force Participation | 0.0555 | -0.00620 | 0.231 | 0.0251 | -0.868*** | -0.0640** | 0.106 | 0.00370 |
|  | (0.136) | (0.0300) | (0.183) | (0.0224) | (0.246) | (0.0244) | (0.249) | (0.0229) |
| Constant | -1.123 | 0.976 | 1.969 | 1.021 | 37.69*** | 4.295*** | -4.959 | 1.422 |
|  | (6.111) | (1.354) | (8.261) | (1.009) | (11.11) | (1.101) | (11.23) | (1.031) |
|  |  |  |  |  |  |  |  |  |
| Observations | 37 | 37 | 37 | 37 | 37 | 37 | 37 | 37 |
| R-squared | 0.665 | 0.620 | 0.616 | 0.537 | 0.608 | 0.542 | 0.686 | 0.640 |

Standard errors in parentheses

*** p<0.01, ** p<0.05, * p<0.1

Table 5SF. Linear regression models of household sugary drink consumption on FREI Informas

|  | (1) | (2) | (3) | (4) | (5) | (6) |
| --- | --- | --- | --- | --- | --- | --- |
| VARIABLES | Soft Drinks - Litres per Household | Soft Drinks - Litres per Household (Logged) | Sports EnergyDrinks - Litres per Household | Sports EnergyDrinks - Litres per Household  (Logged) | Carbonates - Litres per Household | Carbonates - Litres per Household  (Logged) |
|  |  |  |  |  |  |  |
| FREI (Informas) | -100.6** | -0.268** | -1.485 | -0.314 | -49.28*** | -0.375*** |
|  | (40.77) | (0.110) | (2.193) | (0.342) | (17.03) | (0.134) |
| Sub Regions of Europe |  |  |  |  |  |  |
| Central Eastern (Reference) |  |  |  |  |  |  |
| Sub Regions of Central Western | 84.57 | 0.325 | 4.941 | -0.0335 | 30.73 | 0.261 |
|  | (96.81) | (0.262) | (5.207) | (0.812) | (40.43) | (0.318) |
| Eastern | -154.7** | -0.527** | -4.275 | -1.674*** | -6.949 | -0.138 |
|  | (71.62) | (0.193) | (3.852) | (0.601) | (29.91) | (0.235) |
| Nordic | -92.21 | -0.177 | 0.998 | 0.0584 | 29.53 | 0.233 |
|  | (81.28) | (0.220) | (4.372) | (0.682) | (33.94) | (0.267) |
| South Eastern | 59.22 | 0.181 | -1.274 | -0.373 | 22.99 | 0.158 |
|  | (58.91) | (0.159) | (3.168) | (0.494) | (24.60) | (0.193) |
| Southern | 96.38 | 0.251 | 0.102 | -0.390 | -4.598 | -0.0826 |
|  | (69.77) | (0.189) | (3.753) | (0.585) | (29.14) | (0.229) |
| Western | 56.52 | 0.162 | 7.342* | 0.439 | 13.49 | 0.0876 |
|  | (71.74) | (0.194) | (3.859) | (0.602) | (29.96) | (0.235) |
| GDP Annual Growth (%) | 4.607 | 0.00913 | 0.678** | 0.0824* | 6.228*** | 0.0392** |
|  | (5.169) | (0.0140) | (0.278) | (0.0434) | (2.159) | (0.0170) |
| Urban Population % of Total Population | 4.580** | 0.00927 | 0.111 | 0.0221 | 2.342** | 0.0172** |
|  | (2.061) | (0.00557) | (0.111) | (0.0173) | (0.861) | (0.00676) |
| Female Labor Force Participation (%) | -8.290 | -0.00774 | 0.522 | 0.0717 | 2.240 | 0.0149 |
|  | (6.264) | (0.0169) | (0.337) | (0.0525) | (2.616) | (0.0206) |
| Constant | 587.1** | 5.966*** | -22.69 | -2.504 | -87.48 | 3.324*** |
|  | (281.3) | (0.760) | (15.13) | (2.359) | (117.5) | (0.923) |
|  |  |  |  |  |  |  |
| Observations | 37 | 37 | 37 | 37 | 37 | 37 |
| R-squared | 0.657 | 0.622 | 0.543 | 0.536 | 0.479 | 0.445 |

Standard errors in parentheses

*** p<0.01, ** p<0.05, * p<0.1

Table 6SF. Linear regression models of sugary confectionery and savoury snacks consumption on FREI Informas scores

|  | (1) | (2) | (3) | (4) | (5) | (6) | (7) | (8) |
| --- | --- | --- | --- | --- | --- | --- | --- | --- |
| VARIABLES | Sugar Confectionery - Kgs per Household | Sugar Confectionery - Kgs per Household (Logged) | Choco Confectionery- Kgs per Household | Choco Confectionery- Kgs per Household (Logged) | Biscuits- Kgs per Household | Biscuits- Kgs per Household (Logged) | Savoury Snacks - Kgs per Household | Savoury Snacks - Kgs per Household (Logged) |
|  |  |  |  |  |  |  |  |  |
| FREI (Informas) | 0.0675 | 0.116 | -1.800 | -0.217 | -0.108 | -0.0831 | -1.907 | -1.907 |
|  | (0.896) | (0.198) | (1.189) | (0.146) | (1.628) | (0.162) | (1.642) | (1.642) |
| Sub Regions of Europe |  |  |  |  |  |  |  |  |
| Eastern Central |  |  |  |  |  |  |  |  |
| Central Western | 1.896 | 0.287 | 5.052* | 0.406 | 7.483* | 0.900** | 9.752** | 9.752** |
|  | (2.126) | (0.470) | (2.822) | (0.347) | (3.866) | (0.384) | (3.898) | (3.898) |
| Eastern | 4.812*** | 0.814** | -3.581* | -0.488* | 1.663 | 0.0759 | -2.039 | -2.039 |
|  | (1.573) | (0.348) | (2.088) | (0.257) | (2.860) | (0.284) | (2.884) | (2.884) |
| Nordic | 5.879*** | 0.854** | 2.428 | 0.128 | -0.512 | 0.216 | 10.09*** | 10.09*** |
|  | (1.785) | (0.395) | (2.369) | (0.292) | (3.246) | (0.322) | (3.273) | (3.273) |
| South Eastern | -1.034 | -0.472 | -2.650 | -0.218 | 1.713 | 0.306 | 1.522 | 1.522 |
|  | (1.294) | (0.286) | (1.717) | (0.211) | (2.352) | (0.233) | (2.372) | (2.372) |
| Southern | -0.314 | -0.324 | -4.323** | -0.656** | 4.165 | 0.447 | 4.738 | 4.738 |
|  | (1.533) | (0.339) | (2.034) | (0.250) | (2.786) | (0.277) | (2.809) | (2.809) |
| Western | 3.919** | 0.701* | 4.147* | 0.199 | 1.701 | 0.350 | 2.090 | 2.090 |
|  | (1.576) | (0.348) | (2.091) | (0.257) | (2.865) | (0.284) | (2.889) | (2.889) |
| GDP Annual Growth (%) | 0.154 | 0.0172 | 0.266* | 0.0168 | 0.0128 | -0.00128 | 0.637*** | 0.637*** |
|  | (0.114) | (0.0251) | (0.151) | (0.0186) | (0.206) | (0.0205) | (0.208) | (0.208) |
| Urban Population % of Total Population | 0.0257 | 0.00591 | -0.00710 | 0.00612 | 0.162* | 0.0107 | 0.130 | 0.130 |
|  | (0.0453) | (0.0100) | (0.0601) | (0.00740) | (0.0823) | (0.00817) | (0.0830) | (0.0830) |
| Female Labor Force Participation | 0.0509 | -0.00814 | 0.263 | 0.0280 | -0.870*** | -0.0638** | 0.123 | 0.123 |
|  | (0.138) | (0.0304) | (0.183) | (0.0225) | (0.250) | (0.0248) | (0.252) | (0.252) |
| Constant | -0.989 | 1.050 | 0.771 | 0.902 | 37.72*** | 4.272*** | -5.802 | -5.802 |
|  | (6.179) | (1.366) | (8.199) | (1.010) | (11.23) | (1.115) | (11.33) | (11.33) |
|  |  |  |  |  |  |  |  |  |
| Observations | 37 | 37 | 37 | 37 | 37 | 37 | 37 | 37 |
| R-squared | 0.665 | 0.622 | 0.630 | 0.547 | 0.608 | 0.541 | 0.687 | 0.687 |

Standard errors in parentheses

*** p<0.01, ** p<0.05, * p<0.1

**Methodological approach used in the GNPR2 Survey**

Our study includes the results for the WHO European Region, where the questionnaire was sent to national focal points for NCDs within the Ministry of Health in 53 Member States. The questionnaire was available in English, French, Russian and Spanish and designed in an online format.

The GNPR2 contained 42 top-level questions, with additional sub-sections. While the questionnaire was very comprehensive, our study focuses only on the indicators from the GNPR2 that provided information about the food environment policies in countries^^[[1]](#footnote-1)^^. These indicators correspond to the policy subcategories included in the FREI . A total of 50 countries out of 53 responded to the questionnaire, corresponding to 94% of the WHO European Region Member States. Survey answers were compiled into a database, which was subsequently made available online^^[[2]](#footnote-2)^^.

**Table 1: Objectives and main actions recommended in the WHO European Food and Nutrition Action Plan**

| **Objective** | **Main actions** |
| --- | --- |
| 1. Create healthy food and drink environments. | 1.1. Facilitate healthier food choices in schools, including setting standards for the foods available.  1.2. Promote the use of easy-to-understand or interpretative, consumer-friendly labelling on the fronts of packages and healthy retail environments.  1.3. Improve the nutritional quality of foods and beverages available in supply, through product reformulation including salt reduction programmes and the ban or virtual elimination of trans fatty acids from the food supply.  1.4. Adapt measures to reduce the overall impact on children of all forms of marketing of foods high in energy, saturated fats, trans fatty acids, sugar or salt, including through nutrient profiling.  1.5. Implement targeted fiscal measures to influence diets, considering their impact on vulnerable groups. |
| 2. Promote the gains of a healthy diet throughout life, especially for the most vulnerable groups. | 2.1 Promote healthy diet and nutrition before conception, including the provision of nutrition recommendations related to preconception, pregnancy and post-partum.  2.2 Increase measures to protect, promote, support and address barriers to adequate breastfeeding, and provide appropriate complementary feeding; Adopt national guidelines, in addition to monitoring and establishing standards for the marketing of complementary foods, counselling on exclusive breastfeeding as per WHO recommendations^22^; Implement the International Code of Marketing of Breast-milk Substitutes (the Code) and the Baby-Friendly Hospital Initiative (or similar standards) and include comprehensive monitoring of these.  2.3 Consider strategic communication with the public to improve the ability of citizens to make healthy choices, taking into account the needs of different age groups, genders and socioeconomic groups. This can include education on nutrition and health diets, media campaigns, dietary guidelines, the use of social media and new techniques to promote healthy food choices and healthier lifestyles. |
| 3. Reinforce health systems to promote healthy diets. | 3.1 Provision of education and counselling on nutrition and healthy diets for prevention of overweight, obesity and diet-related noncommunicable diseases in primary health care, including elements of behaviour change and considering effective measures to reach at-risk groups.  3.2 Improve nutrition capacity and training for relevant health professionals to enable the provision of high quality nutrition services in health care settings. |
| 4. Support surveillance, monitoring, evaluation and research. | 4.1 Strengthen and expand nationally representative diet and nutrition surveys and to ensure the availability of anthropometric data, particularly for children under five years.  4.2 Establish and maintain food consumption databases and anthropometric surveillance systems that allow disaggregation by socioeconomic status and gender. |

1. ^Indicators on other nutrition issues, such as management of acute malnutrition, were also collected but are considered beyond the scope of this paper.^ [↑](#footnote-ref-1)
2. https://gateway.euro.who.int/en/datasets/gnpr-survey/ [↑](#footnote-ref-2)
